# Entrapment of water by subunit c of ATP synthase

**DOI:** 10.1098/rsif.2007.1146

**Published:** 2007-09-11

**Authors:** Julie E.M McGeoch, Malcolm W McGeoch

**Affiliations:** 1 Department of Molecular and Cellular Biology, Harvard University7 Divinity Avenue, Cambridge, MA 02138, USA; 2 PLEX LLC275 Martine Street, Fall River, MA 02723, USA

**Keywords:** proteolipid, encapsulating water, 4 Gyr ago

## Abstract

We consider an ancient protein, and water as a smooth surface, and show that the interaction of the two allows the protein to change its hydrogen bonding to encapsulate the water. This property could have made a three-dimensional microenvironment, 3–4 Gyr ago, for the evolution of subsequent complex water-based chemistry. Proteolipid, subunit c of ATP synthase, when presented with a water surface, changes its hydrogen bonding from an α-helix to β-sheet-like configuration and moves away from its previous association with lipid to interact with water surface molecules. Protein sheets with an intra-sheet backbone spacing of 3.7 Å and inter-sheet spacing of 6.0 Å hydrogen bond into long ribbons or continuous surfaces to completely encapsulate a water droplet. The resulting morphology is a spherical vesicle or a hexagonal crystal of water ice, encased by a skin of subunit c. Electron diffraction shows the crystals to be highly ordered and compressed and the protein skin to resemble β-sheets. The protein skin can retain the entrapped water over a temperature rise from 123 to 223 K at 1×10^−4^ Pa, whereas free water starts to sublime significantly at 153 K.

## 1. Introduction

When considering the mechanisms that allowed complex chemistry to start in the biospheres, which happened on Earth after water started to condense 4.4 Gyr ago (Wilde *et al*. 2001), the nature of the water vessel is of the utmost importance. Mineral compartments including FeS and NiS are proposed for hosting the 3.8 Gyr ago pre-cell state and supplying catalytic surfaces (Martin & Russell 2003, 2006), but a system in the period before this, and also later when the first cells emerged, had to assist the first chemistry by retaining water in micro-vessels. The ideal vessel would be versatile so that when water was abundant it had a labile form that could spread/grow/diffuse into other environments but when water was evaporating it could encapsulate the water and resist its evaporation. It would also address the need to concentrate the basic reactants (Duve & Miller 1991; Baaske *et al*. 2007). There is one ancient 7–8 kDa protein that is the most conserved through all organisms, which, together with its associated lipid, could possibly be shown now to have a water vessel role, a role which could therefore have been shared 3–4 Gyr ago with its protein ancestors and the simple lipids of the time. Subunit c of the ATP synthase is highly conserved back to Archaea (Coskun *et al*. 2004), the ancestors of which first fixed carbon dioxide (Mojzsis *et al*. 1996) alongside Eubacteria and pre-cells 3.8 Gyr ago. By light microscopy, we became very familiar with the forms that this protein adopts in a variety of solvents including water, while attempting to solve its three-dimensional structure by X-ray crystallography. A solution of pure subunit c and phospholipids in organic solvent when placed in water on a glass surface grows out into hollow vesicles and tubes, essentially engulfing and partitioning water (figure 1). The protein in the same solution conditions, when painted over barrier holes of 30–1000 nm diameter, assembles into cation-conducting channels (McGeoch & Guidotti 1997; McGeoch *et al*. 2000). The vesicles/tubes of figure 1 are likely to have had the protein in a channel configuration because they grew in volume as though in response to inward conduction of water, leading to stretching and elongation away from their original position. The conductance of the subunit c channel is tightly regulated by calcium (McGeoch & McGeoch 1994; McGeoch & Palmer 1999), so in pure water it will tend to open with large conductance and transport water. To further investigate the physical association of water and the proteolipid in the vesicles/tubes, we examined the phenomenon on a sub-micron to nanometre scale by Cryo-SEM and Cryo-TEM microscopy and analysed the central water by electron diffraction.

We feel justified to use a protein rather than single amino acids in our mimicking of early conditions 3–4 Gyr ago, as laboratory chemical simulation experiments of the predicted conditions 3–4 Gyr ago yield many compounds and experimental time seems the only limit to producing almost any compound (Cody *et al*. 2000). Therefore, chance would have produced sufficient opportunities for sugars, lipids, amino acids and more complex chemicals to form. Lastly, proteins are seen to form readily from amino acids in, for example, lipid domain boundaries (Raine & Norris 2007) and in situations where nanometre scale conjunction condenses the amino acids (Zhang 2003). That these events also were occurring or had occurred in many biospheres and could have contributed chemicals to Earth via extraterrestrial in-fall is summarized in electronic supplementary material, slide 1, where the analysis of comet and meteorite material is presented. The present work relates to Earth-like conditions of pressure and temperature, with liquid water.

## 2. Methods

### 2.1 Materials

Water was deionized to 19 MΩ and the 75 amino acid mammalian subunit c (Dyer & Walker 1993) purified by methods already described. Essentially, the purification involved preparing membranes from brain or using commercially prepared plant subunit c in phosphatidyl ethanolamine and phosphatidyl serine. The brain membranes were chloroform–methanol extracted to leave in the chloroform phase subunit c and its associated lipids. Its purity was indicated by a single protein band on sodium dodecyl sulphate-polyacrylamide gel electrophoresis, which gave the correct amino acid sequence from automated polypeptide sequencing using standard phenyl isothiocyanate reagent and HPLC analysis (McGeoch & Guidotti 1997). Further ether precipitation of the protein from the chloroform phase with resolubilization in chloroform removed more lipid but never all the lipid, and adding mass- and ion-based chromatography column separation techniques after this still resulted in subunit c associated with a significant amount of lipid. The identity of the lipid component by silica gel chromatography was phosphatidyl serine, phosphatidyl ethanolamine, phosphatidyl choline and cholesterol.

At atmospheric pressure and room temperature, solutions of subunit c (1–10 μg ml^−1^ in chloroform/methanol/water 70/25/5) were mixed with 2–10× as much water, on glass coverslips, aluminium SEM stubs and copper TEM grids (backed with Formvar resin, overlaid with 0.3 nm thick lacey carbon islands in the grid wells). On the glass coverslips, the morphologies could be imaged in the presence of water that had not undergone evaporation, but for the SEM and TEM samples, where micron to nanometre detail was sought, the samples were flash-frozen at a point when no liquid water was visible around the sample and less than 10 s had elapsed following complete evaporation. This process was monitored under 25× magnification on a bench zoom light microscope with the TEM grid held in locking forceps, or the SEM stub on the microscope stage. These samples went from room temperature to 78 K in milliseconds and into a vacuum of 1×10^−4^ Pa (SEM) or 1×10^−6^ Pa (TEM). Some of the SEM samples were sputter coated, after freezing and at 1×10^−4^ Pa, with 4 nm gold (Au) to avoid charging when imaging.

### 2.2 SEM instrument conditions

The SEM was performed using an FEI 235 dual-beam electron microscope (Eindhoven, The Netherlands) with a Gatan Alto 2500 cold chamber, and stage (Oxford, UK). The electron beam alone was employed for this research. Experiments were conducted, unless otherwise mentioned, between 93 and 123 K. The electron beam source was a field emission gun at 5 kV at a vacuum of 1×10^−4^ Pa. The electron beam minimum spot size was 2 nm at 1 pA on a silicon substrate. The images are formed from secondary electrons entering the instrument collectors/detectors.

### 2.3 TEM instrument conditions

The TEM was performed using a Jeol JEM-2100F electron microscope (Tokyo, Japan). The TEM beam energy was 200 keV at a vacuum of 1×10^−6^ Pa, and the initial stage temperature was 83 K. The TEM parameters for detecting diffraction were calibrated just prior to each experiment with nickel oxide. X-ray diffraction patterns from three separate directions were taken of the hexagonal water crystals surrounded by proteolipid and spherical vesicles of the same.

## 3. Results

A solution of subunit c mixed with water forms one dense droplet or several, due to the 1.56 density of its chloroform solvent relative to 1 for water. The chloroform starts to evaporate and from all the droplets cylindrical tubes with visible central lumens grow out within a minute into the water (figure 1). If the sample has had more lipid removed relative to the protein via an ether precipitation step (see section 2.1 Materials), then fewer tubes grow and they are shorter because lipid with the protein is needed for the tube formation. If the water evaporation is controlled, by placing a glass coverslip over the preparation, within days cylindrical hollow crystals (figure 1, bottom right inset) and hexagonal crystals grow. Even if all the water is allowed to evaporate and the preparation essentially dries on the slide, rehydration with water will produce some tube growth from dried areas of lipid and protein, from cylindrical crystals, but not from hexagonal crystals. In water, the terminal ends of the tubes frequently pinch off into vesicles (figure 1, top right), and thin tubes less than 1 μm in diameter fold and twist back on themselves (figure 1, top left inset). The angstrom detail of the tube walls is discussed later in figure 7 via a TEM analysis of the strands and channels of protein within the walls.

SEM images show more external detail of the long hollow crystals with hexagonal ends (figure 2*a*) and branching tubes amidst many small crystals and vesicles (figure 2*b*–*d*). The formation of the vesicles via encapsulation by subunit c can be very uniform and produce a monolayer of almost identically sized vesicles across the stub, aligned to any straight groove on the Al surface (figure 3). The Al SEM stubs come from the manufacturer with fine straight sub-micron grooves from polishing. This perfect alignment of the vesicles came when they were warmed slowly, at 2 K min^−1^, from 83 to 233 K within the SEM instrument. Their central water remained as ice up to 223 K and did not sublime. Free water ice would have begun to sublime significantly (1 nm/100 s is significant for our experiments) at 153 K. Between figure 3*a* and figure 3*c*, there was a flattening of the vesicles and hints of hexagonal form are apparent in figure 3*c*. Between figure 3*c* and figure 3*d*, the temperature rose from 223 to 233 K and the water in the vesicles exited abruptly causing a pressure transient to 1×10^−4^ Pa, just sufficient to trigger a temporary ‘shut down’ of the imaging. Possibly, hydrogen bond configurations between the vesicle central water and protein abruptly changed between 223 and 233 K. Although this event occurred below 273 K, mobility of water is known to be possible well below 273 K as the result of ordering by the proximity of protein hydrogen bonds (Weik 2003). Transitions from a locked to a mobile state can occur over the span of as little as 10 K, an example being the purple membrane water system which transitions abruptly to mobility above 255 K (Weik 2003). In figure 3*d*,*e*, the instrument is recooling down to 83 K, and imaging is again possible, but during cooling the vesicles do not undergo further change to their morphology in terms of size or shape. The vesicle morphology is, however, interesting. Each cluster is 42 times larger than at the beginning of the warming process (compare figure 3*a* with figure 3*d*,*e*). When the central water liquefied, the subunit c skin must have flowed around the escaping water to produce flattened spheres that flopped over one another. The vesicle clusters then freely diffused across the SEM stub aligning to any indent, and a few clusters that could not fit across the stub made second-layer projections in discrete places (figure 3*e*).

The TEM images reveal that the water morphology is a sphere or a hexagonal crystal that is encapsulated by a skin of subunit c. Spacious vesicles around hexagonal ice crystals (figure 4*a*), vesicles whose sides are beginning to flatten as they abut part of a crystal edge (figure 4*b*), vesicles joined to crystals (figure 4*c*) and vesicles and crystals attached to one another where each morphology is in transition (figure 4*d*) are present. Basically, the skin of subunit c winds around the water (figure 5*a*). It is necessary at this point to define the terms used to describe the TEM imaged subunit c morphologies. The smallest unit of subunit c is termed a ‘strand’ and is the carbon backbone of the protein, which is electron dense due to its planar-amide bonds. ‘Sheets’ are composed of 17–24 laterally spaced strands with a lateral spacing of 3.7 Å (figure 5*b*), connected inter-molecularly by β-sheet-type hydrogen bonds. A ‘ribbon’ is a very long sheet that can consist of one or several sheet layers and extend continuously for hundreds of nanometres. The edges of the ribbons that face away from the vesicle body have an area of α-helices, and therefore have both β-sheet and α-helix hydrogen bonding in the subunit c protein. The ribbon of subunit c that runs diagonally across in figure 5*b* is from the right-side edge of the vesicle in figure 5*a*, in which the outer sheet structure is more loosely wound, allowing clear imaging. It has 24 strands laterally, and lengthways the alignment goes across the image for greater than 134 nm. Going further left into the vesicle, criss-crossing ribbons can be seen. Images of the ribbons cladding the vertical edges of crystals of water, perpendicular to the flat ribbon direction above, show that the spacing is 6 Å between the sheet planes (figure 6*d*). The same image shows that the ribbons of subunit c around the crystal bend to accommodate the 60° angle turn.

The water crystals encapsulated by the skin of subunit c ribbons are highly ordered (figure 6). Figure 6*b*(i) shows the ‘abandoned’ lipid in the sample lying as pale puffy accumulations among the darker electron-dense hexagonal water crystals surrounded by subunit c. Even with short exposure, to minimize damage by the beam, the lipid does not show evidence of regular structure and does not diffract, while the water crystals show almost perfect hexagonal water ice diffraction patterns, only degrading after many seconds of exposure. The spacing of the first peaks from the centre of the diffraction pattern in three separate crystals, each measured from one to three directions, corresponded to a lattice spacing of 3.73±0.06 Å, *n*=36. This measurement is a little smaller than that expected for hexagonal water ice of 3.9 Å (Barnes 1929; Dowell & Rinfret 1960; Goto *et al*. 1988). In Dowell & Rinfret (1960), the X-ray diffraction measurement was performed at 98 K, very close to our experimental temperature of 93 K, and the corresponding parameter was 3.897±0.002 Å. Our measured lattice of 3.73 Å lies between the strongest lattice reflection of hexagonal ice at 3.897 Å and the shared lattice spacing of cubic and hexagonal ice at 3.66 Å (Dubochet *et al*. 1988; Murray & Bertram 2006). Two additional factors lead us to identify the structure as hexagonal. The first is the sixfold symmetry axis of the patterns (hexagonal ice has Laue group 6*mmm*), which, although not perfect in terms of intensity, is very clear. Cubic ice can exhibit only a threefold symmetry axis (Laue group $\bar{m}$3*m*). The second is the growth habit of our crystals, which is equal in all six directions, and completely identifiable with that of hexagonal ice.

Another morphology of very narrow tubes of subunit c is also seen on the TEM grids (figure 7). The tubes are in collapsed piles with crystals in the centre of the pile (figure 7, inset). The surface of the tubes has strands of protein with the typical 3.7 Å spacing, and between the strands are circular entities of 3 nm diameter that could be channels of subunit c. A 10-mer channel would have a diameter of approximately 3 nm. This morphology is expected to contain protein and lipid around the central water within the tube.

## 4. Discussion

Subunit c is an ancient protein and every organism on Earth today has a similar amino acid sequence, with all mammals analysed to date showing the same sequence precisely (Dyer & Walker 1993; electronic supplementary material, slide 2). One may therefore predict that the ancient chemical systems, the precursors of the Archaea and Eubacteria and other pre-cell states present 3–4 Gyr ago, were also starting to acquire proteins similar to subunit c. Subunit c emerges as an extremely versatile molecule. It functions alone as a cation channel capable of generating an oscillating current through a polarized membrane (McGeoch & Guidotti 1997), and as a rotor within the ATP synthase complex (Senior *et al*. 2002). In each of these roles, there is evidence that it is configured as an α-helical hairpin, grouped in a ring of many subunits to form a channel. In prior work we had noted its ability, in the presence of some lipid, to rapidly form tubes and vesicles when presented to water, and in the present work the end products of this process, after dehydration, were found to be water-filled vesicles encapsulated by ribbons containing sheets of subunit c in the β-configuration.

Electron diffraction patterns of extremely high resolution of the hexagonal water crystals encased by ribbons of subunit c indicate the water to be slightly compressed as well as highly ordered. The protein itself is unlikely to be giving rise to the diffraction patterns although the inter-backbone spacing is similar to the water lattice within our experimental accuracy. The protein strands are ordered and easily seen on the angstrom level with the electron wavelength of 2.5 pm, but to obtain the sharp diffraction spots seen in the crystals they would have to be aligned in a regular array to within 1 pm and this is unlikely for any protein, particularly a membrane protein.

The specimen had been rapidly plunged into liquid nitrogen after hydration of the lipid/protein sample, cooling from room temperatures of 16–22°C to 78 K within milliseconds, so water that might have been trapped within vesicles would have been expected to form ‘vitreous’ ice, comprising a disordered glassy assembly with the possible presence of very small, randomly oriented ice crystals of the cubic or hexagonal form (Blackman & Lisgarten 1957; Dowell & Rinfret 1960; Shimaoka 1960). Between the moment of freezing and taking of the diffraction measurement, the temperature did not rise above 93 K, whereas the transition from vitreous to cubic only begins to occur, and even then very slowly, when the temperature is raised to the 113 (Dowell & Rinfret 1960) or 123 K (Shimaoka 1960) range. The further transition from cubic to hexagonal occurs only above 143 K (Dowell & Rinfret 1960). It seems difficult to avoid the conclusion that the hexagonal ordering could only have occurred prior to freezing, during preparation of protein/lipid vesicles at room temperature.

The structure of water at room temperature has been studied in neutron diffraction (Soper & Phillips 1986) at 25°C, where it is shown that ‘the O–O distribution has a well-defined peak at 2.975 Å consisting of approximately 4.5 oxygen atoms’ and ‘the near neighbour coordination of water molecules is well-defined and roughly tetrahedral at any instant in time’. In contrast, the hexagonal ice structure has very close to exact tetrahedral coordination with an O–O bond length of 2.58 Å (at 98 K, estimated from Dowell & Rinfret (1960)) in the bonds aligned closest to the *a*-axis. In recent work by Chaplin (1999), a 280 molecule loosely coordinated structure has been proposed to explain many of the properties of liquid water. Liquid water at room temperature is therefore on the verge of being ordered and may possibly be tipped into exact hexagonal ordering by a hydrogen bonding interaction with an appropriate organizing structure on its surface, in this case the β-sheet ribbons of subunit c. Such interactions are beginning to be understood (Cameron *et al*. 1997) but not specifically applied to subunit c and hexagonal water.

The fungal hydrophobin proteins also encapsulate water by forming films and exert a flattening influence on water droplet surfaces (Szilvay *et al*. 2007). However, the hydrophobins are different from subunit c in that they are rich in cysteines, which are involved in their intermolecular bonding, and they have separated regions of both hydrophobic and hydrophilic amino acids (Hakanpää *et al*. 2004), while subunit c is almost entirely hydrophobic and has only one cysteine in the mammalian and yeast sequence and none in *Escherichia coli* (electronic supplementary material). Another difference is that the very long uninterrupted length of the β-backbones in the observed subunit c sheets seems to imply polymerization of subunit c as the β-sheets within a ribbon are formed. However, the edges of sheets often showed the appearance of α-helices (figure 4*b*), indicating a certain fluidity of structure between the two forms. Protein α-helices are often seen to transition to β-sheets. Perutz *et al*. (2002) have postulated the formation of amyloid β-sheet open cylinders surrounding water, and Singer & Dewje (2006) conducted experiments that suggest these structures make channels in membranes. The surface of carbon nanotubes can facilitate a change from an α-helix to β-sheet conformation (Sugiyama *et al*. 2006). In the present work, the hydrophobic subunit c occupies a water surface and may be helped in the α-to-β transition by the underlying hydrogen bond structure of water. In effect, the β-sheet is stabilized as the water becomes more ordered in a *cooperative* process.

The change to a flat entrapping ribbon on the part of a protein could conserve water, and this would be a useful property 3–4 Gyr ago for a micro-vessel holding the first constituents for complex chemistry. We also note that when this protein is associated with lipid it forms tubes that branch and twist (figures 1 and 2). This topology facilitates entry and exit of substrates and allows substrates to be held on catalytic walls in small conduits preventing the products from crystallizing, in contrast with the structure of vesicles in water, which does not allow entry and exit and may not prevent product crystallization. One could speculate that the branching tubes of proteolipid were the forerunner in the pre-cell 3–4 Gyr ago to the endoplasmic reticulum (ER) of Eukaria, particularly as the gene duplication of subunit c, the subunit c of the V-ATPase, is present in the Golgi ER (Flannery *et al*. 2004). We note that the tight twists of the tubes (figure 1, top left inset) are a more extreme property of very narrow tubes of proteolipid, but this phenomenon can also be induced when lipid is extruded from nanoholes, and there, rather than twisting like a rope, it coils into helices (Dittrich *et al*. 2006).

We conclude that an ancient protein, subunit c of ATP synthase, aided by lipid, can expand into water and partition it into tubes and vesicles. On evaporation of the surrounding water, this hydrophobic protein accumulates on the vesicle surface and in contact with water condenses into a dense β-sheet ribbon that is substantially impermeable. Water is thereby encapsulated and conserved in an environment with potentially a large surface area and small free internal volume, of the type conducive to the emergence of complex chemistry. Unexpectedly, our observations open a window into a mechanism that allows a suitably constructed early protein to capture and control its surrounding aqueous space, conferring upon the protein a decisive evolutionary advantage, either on its own or in conjunction with other molecules. Apart from the properties already described for subunit c (as a representative proteolipid protein), three additional factors would be required for its ‘reproductive’ success: a supply, in an aqueous environment, of its constituent amino acids; a supply of lipid to form a matrix for the α-structure ion channel surfaces that allow water and amino acid uptake prior to encapsulation; and a catalytic mechanism to create exact copies of itself via peptide condensation. Of these, the third seems to be the greatest unknown. A direction for further investigation would be to search for peptide condensation at the surface of water, stimulated or guided by a proteolipid protein skin.

## Figures and Tables

**Figure 1:**
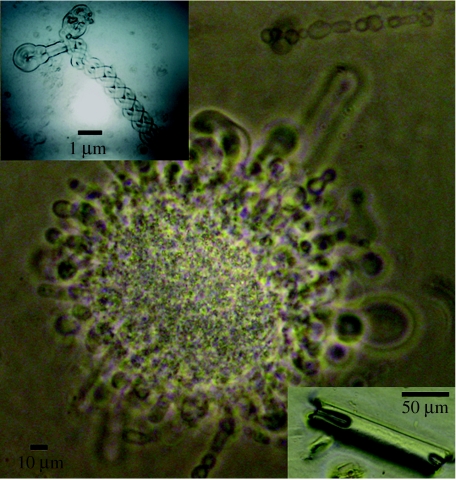
Phase-contrast images of tubes of subunit c. When the volume of solvent above the initial lipid/subunit c droplet is sufficient to allow the tubes to extend without reaching an air/solvent interface a mass of tubes grow out and up from the droplet to form a medusa-like structure. Subunit c tubes at the outer surface of the water droplet (top right) round off and form vesicles. The range of diameter of the tubes is 5–40 μm. If evaporation of the water is considerably slowed then crystals grow that are sometimes hollow (bottom right inset). Long thin tubes fold and twist back (top left inset, inverted phase-contrast image to reveal twist detail).

**Figure 2:**
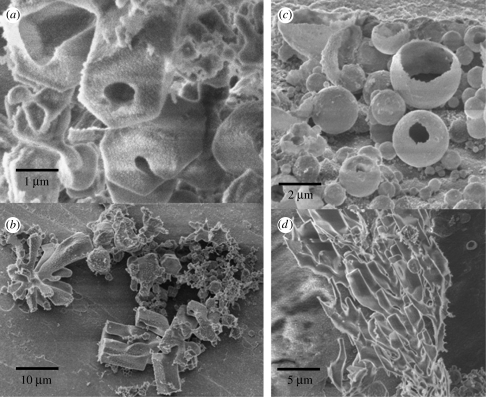
SEM images of the morphologies encountered when subunit c is repeatedly hydrated and dehydrated. (*a*) Hexagonal-ended hollow crystals. (*b*) Branching tubes. (*c*) Spherical vesicles with thick multilayered 300 nm walls. (*d*) Branching tubes.

**Figure 3:**
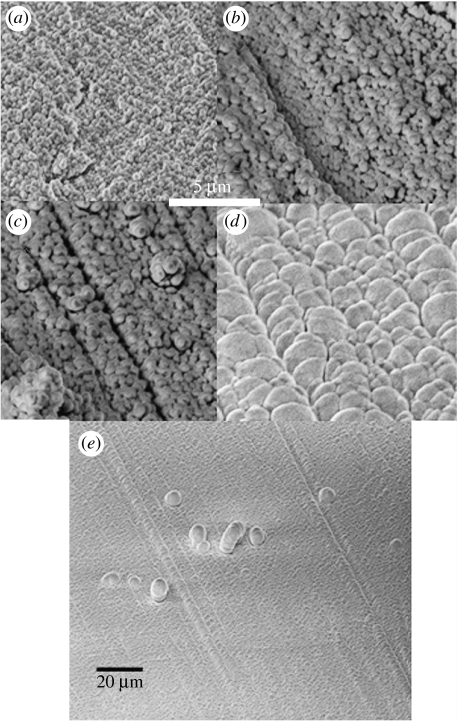
SEM images showing the effect of raising the temperature of subunit c vesicles from 83 to 233 K to sublime the central water. (*a*) Vesicles of subunit c are initially less than 1 μm in diameter on the Al stub at 83 K. (*b*) They expand on warming as their central ice reconfigures. (*c*) At 213 K, each vesicle has expanded further and has a very thin relatively dark wall area. In all experiments once the temperature reaches 233 K, the central water in the vesicles suddenly escapes from each vesicle resulting in the instrument abruptly registering a significant ‘out-gassing or sublimation event’. The instrument vacuum of 1×10^−4^ Pa, however, remains intact but images cannot be captured until the temperature is lowered to halt further sublimation. (*d*) Here the temperature is being lowered again and has reached approximately 123 K. The vesicles are now multiple consisting of several flattened vesicles collapsed around each other to form spheres of approximately greater than 4 μm diameter. They are aligned completely across the stub, having nucleated to an arbitrary sub-micron straight groove on the stub. (*e*) A larger area than (*d*) showing the extent of the alignment of the vesicles. Some grow up above the monolayer and collapse back.

**Figure 4:**
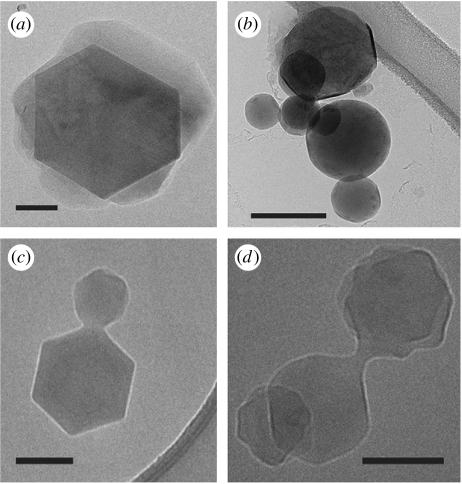
TEM images of transitional forms of subunit c encapsulating water. (*a*) Spacious vesicles with central crystals. (*b*) Vesicles whose sides are beginning to straighten. (*c*) Spherical vesicles with attached hexagon crystals. (*d*) Transitional vesicles and crystals attached to one another. Scale bars, 100 nm.

**Figure 5:**
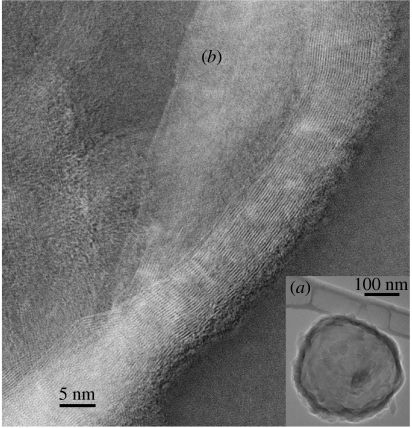
(*a*) TEM image of a vesicle consisting of ribbons of subunit c winding around water. (*b*) TEM *inverted* image of ribbons of subunit c. A ribbon has β-sheets of strands of subunit c laterally spaced 3.7 Å. The edges of the ribbon away from the vesicle are α-helices. Lengthways the strands/carbon backbones of the protein can be followed in the ribbons as continuous strands of length greater than 134 nm diagonally across the entire image. The nearest ribbon has 24 strands with inter-molecular β-sheet-type bonding and α-helices at its right edge. TEM 200 kV, 1×10^−6^ Pa.

**Figure 6:**
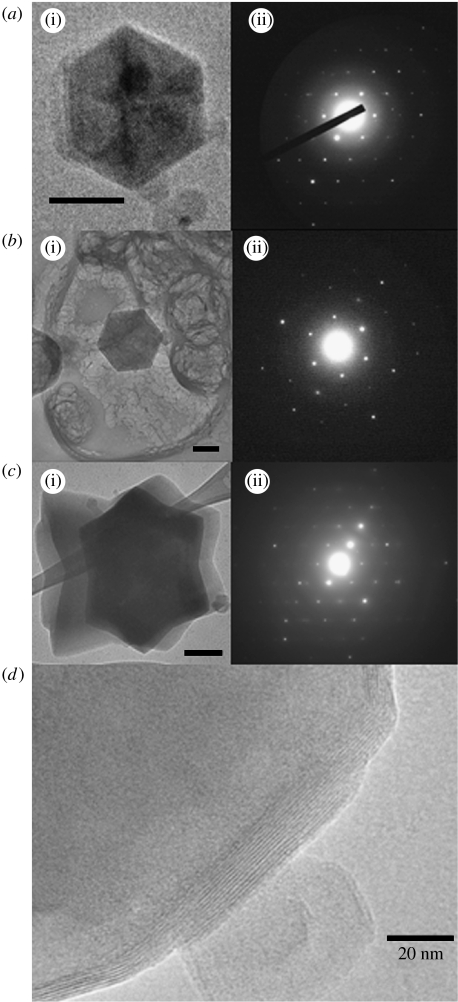
(*a*(i)–*c*(i)) TEM of hexagonal crystals of water encapsulated by subunit c and (*a*(ii)–*c*(ii)) their diffraction patterns; scale bars on (*a–c*) are 100, 100 and 200 nm, respectively. The average spacing from the centre of the pattern to the first hexagonal water spot corresponds to 3.73±0.06 Å in three separate crystals measured from one to three directions. In (*b*(i)), electron-dense spherical vesicles encapsulated by subunit c can be seen together with pale masses of lipid in the background. (*d*) Another hexagon water crystal that shows detail of the subunit c inter-sheet spacing as it encapsulates the crystal. At different points on the edge of this crystal, there are 7–13 separate subunit c sheets spaced 6.0±0.1 Å. The sheets bend over the 60° turns of the crystal.

**Figure 7:**
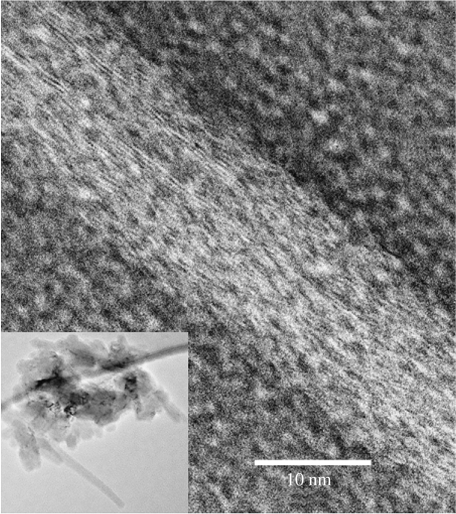
TEM (*inverted image*) of a tube whose surface is covered by strands of subunit c with a lateral spacing of 3.7 Å amidst circular structures that could be assembled channels of subunit c of 3 nm diameter. The strands adopt a woven path over the tube surface. The inset image shows a lower magnification of the entire pile of collapsed tubes on the TEM grid and in their centre are crystals.

## Supplementary Material

S1 entrapment of water by subunit c of ATP synthase A chemical time-line graph; a table of comet and meteorite chemicals; the protein form of *E. coli*, mammal and yeast proteolipid; the predicted mammal, yeast and *E. coli* amino acid distribution at a hydrophobic-hydrophilic interface; ordering of a water droplet by subunit c at room temperature
